# The Nanofibrous CaO Sorbent for CO_2_ Capture

**DOI:** 10.3390/nano12101677

**Published:** 2022-05-14

**Authors:** Vyacheslav V. Rodaev, Svetlana S. Razlivalova, Alexander I. Tyurin, Vladimir M. Vasyukov

**Affiliations:** Institute for Nanotechnology and Nanomaterials, Derzhavin Tambov State University, Internatsionalnaya Str. 33, 392000 Tambov, Russia; razlivalova8@yandex.ru (S.S.R.); tyurin@tsu.tmb.ru (A.I.T.); space-1985@mail.ru (V.M.V.)

**Keywords:** CaO nanofibers, electrospinning, microstructure, phase composition, chemisorption, CO_2_-uptake capacity, mechanical properties

## Abstract

The nanofibrous CaO sorbent for high-temperature CO_2_ capture was fabricated by the calcination of electrospun composite filaments containing calcium acetylacetonate and polyacrylonitrile as a calcium-oxide precursor and a binder polymer, respectively. The calcination was carried out in air to prevent PAN carbonization and to obtain pure CaO nanofibers. The resulting mats of CaO nanofibers with the average diameter of 130 nm were characterized by a specific surface area of 31 m^2^/g, a CO_2_-uptake capacity of 16.4 mmol/g at the carbonation temperature of 618 °C, a hardness of 1.87 MPa, and the indentation Young’s modulus of 786 MPa. The low decarbonation temperature makes the fabricated sorbent promising, for example, for the calcium-looping technology of CO_2_ removal from the hot exhaust gases of fossil-fueled power plants.

## 1. Introduction

Carbon dioxide is considered the main greenhouse gas. In May 2021, the Mauna Loa Observatory registered a CO_2_ concentration of 420 ppm in the atmosphere, and it continues to increase at a rate of about 2 ppm/year. This dynamic could lead to global warming of 2 °C by the end of the 21st century [1]. It is expected that the atmospheric CO_2_ concentration will continue to rise over the next few decades as fossil fuels will still be the dominating energy source. Fossil-fueled power plants are major contributors among the anthropogenic sources of CO_2_ emissions [2]. Nowadays, several methods are used for industrial CO_2_ capture from gas streams, namely absorption by amine solutions, physical adsorption and chemical adsorption [3]. However, amine scrubbing is characterized by a low absorption temperature, a significant energy consumption for regeneration, equipment corrosion and a negative environmental impact. Microporous and mesoporous adsorbents such as activated carbon, carbon nanofibers, graphene, zeolites, metal–organic frameworks and silica also capture CO_2_ at low temperatures, and high CO_2_-uptake capacity is usually achieved if adsorption occurs at elevated pressure. Additionally, these adsorbents are not highly selective for CO_2_, which often requires preliminary purification of the gas stream. For purification of hot exhaust gases from CO_2_ at a pressure close to atmospheric calcium oxide, lithium zirconate and lithium silicates with high selectivity to CO_2_ are used, of which CaO and Li_4_SiO_4_ are cheaper than Li_2_ZrO_3_ due to cost-effective precursors [4,5]. CaO is characterized by a higher stoichiometric CO_2_-uptake capacity compared to Li_4_SiO_4_ (17.9 mmol/g and 8.3 mmol/g, respectively) and better CO_2_ sorption/desorption kinetics. The ability of CaO to reversibly sorb CO_2_ is used in the calcium-looping technology, which is considered a promising approach for a large-scale post-combustion CO_2_ capture [6].

To improve CaO reactivity, various techniques are employed to decrease the particle size and increase the surface area of CaO, including the precipitation reaction [7], the sol-gel method [8], template synthesis [9], flame-spray pyrolysis [10], wet mixing [11], mechanical milling [12], etc.

Electrospinning is a simple, cost-effective, versatile and efficient technique to fabricate nanostructured fibrous materials with a macroporous structure, which provides efficient gas transport to be required for filters, sorbents, and catalysts [13,14]. Electrospun nanomaterials (polymer, metal oxide, carbon, etc.) are being applied to gas separation, to the removal of volatile organic compounds, nanoparticles and airborne bacterial contaminates from air, and to the removal of toxic pollution from wastewater, such as organic dye solutions, heavy metals, phenolic compounds, etc. [15,16,17]. Previously, electrospinning was successfully used to fabricate low-temperature CO_2_-adsorption membranes consisting of carbon nanofibers, polymer nanofibers loaded with metal–organic frameworks, and amine-functionalized polymer nanofibers [18,19,20].

The aim of this work was to use the electrospinning method to obtain nanostructured CaO sorbent for high-temperature CO_2_ capture and to study the morphology and functional characteristics of the resulting nanofibrous material.

## 2. Materials and Methods

A polymer solution of 10 wt.% was prepared by dissolving 1 g of polyacrylonitrile with a molecular weight of 150,000 (PAN, Sigma-Aldrich, Saint Louis, MO, USA) in 9 g of N,N-dimethylformamide (DMF, Sigma-Aldrich, Saint Louis, MO, USA) under magnetic stirring for 2 h at 50 °C. A quantity of 0.5 g of calcium acetylacetonate (CaAA, Sigma-Aldrich, Saint Louis, MO, USA) was added into the fabricated solution and stirred at 50 °C until transparent solution was obtained.

The obtained composite solution was poured into a plastic syringe and then electrospun through a 23 G blunt-tip needle on a rectangular-frame collector made of copper wire. The collector was placed in a NANON-01A electrospinning apparatus (MECC, Fukuoka, Japan). The fibers were collected as non-woven mats. The following electrospinning parameters were used to fabricate smooth and bead-free composite fibers: distance between the needle tip and the collector of 12 cm, the accelerating voltage of 14 kV and a feeding rate of 0.8 mL/h.

To prepare the CaO nanofibers, the electrospun composite filaments were annealed in a muffle furnace at 800 °C for 1 h in air atmosphere. The heating rate was 1 °C/min to ensure the delicate removal of CaAA and PAN decomposition products. The annealing temperature value was chosen in accordance with the results of the thermogravimetric (TG) analysis. The TG analysis was performed on the thermal analyzer EXSTAR TG/DTA7200 (SII Nano Technology, Tokyo, Japan) in air atmosphere with a heating rate of 10 °C/min.

A thermal analyzer was also used to measure the CO_2_-uptake capacity of CaO nanofibers. As-fabricated CaO nanofibers were heated up to the target carbonation temperature at a rate of 10 °C/min and held for 30 min in the gaseous stream containing 15 vol.% CO_2_ and 85 vol.% N_2_. The carbonation occurred at 15 vol.% CO_2_ in the gas flow since such a concentration corresponds to the typical CO_2_ content in the exhaust gases of coal-fired power plants [21]. After carbonation, the filaments were heated up to 800 °C at a rate of 20 °C/min and held for 20 min under N_2_ flow to be decarbonated. The CO_2_-uptake capacity of CaO nanofibers was determined by the amount of captured CO_2_ divided by the sample weight before carbonation. The target carbonation temperature was determined using differential thermogravimetric (DTG) analysis as the temperature when the carbonation rate was maximal. The DTG analysis was performed in the gaseous stream containing 15 vol.% CO_2_ and 85 vol.% N_2_. The DTG analysis in a nitrogen atmosphere was also used to determine the temperature of the maximum decarbonation rate.

The XRD pattern in the 2θ range 20–70° was recorded by a D2 Phaser X-ray diffractometer (XRD, Bruker AXS, Karlsruhe, Germany) using CuKα1 monochromatic radiation. The registered XRD pattern was identified by means of the PDF-2 Diffraction Database File compiled by the International Centre for Diffraction. The phase content was determined from the XRD pattern by the Rietveld method in the TOPAS software (Bruker AXS, Karlsruhe, Germany) and the average grain size was calculated using the Scherrer equation, also in the TOPAS software (Bruker AXS, Karlsruhe, Germany). The diameter and microstructure of the nanofibers were analyzed using a JCM-7000 scanning electron microscope (SEM, Jeol, Tokyo, Japan). XRD and SEM measurements were carried out at room temperature. The specific surface area and specific pore volume of the fibers were calculated from the nitrogen adsorption isotherm obtained at −196 °C using an Autosorb iQ-C gas sorption analyzer (Quantachrome Instruments, Boynton Beach, FL, USA). The specific surface area was calculated by the Brunauer–Emmett–Teller method. The specific pore volume was calculated from the amount of nitrogen adsorbed at the relative pressure of 0.99.

A TI-950 nanotriboindenter (Bruker AXS, Karlsruhe, Germany) was used to determine the hardness and Young’s modulus of nanofiber mats. Mechanical tests were carried at room temperature. A zirconia spherical indenter had a radius of curvature many times larger than the size of the macropores formed by randomly arranged nanofibers. The hardness and the indentation Young’s modulus of the nanofiber mats were calculated from the load–displacement curve using the Oliver–Pharr method. The tested samples were carefully cut from annealed mats and then fixed with ethanol on the polished surface of zirconia ceramic pellets, with the hardness and Young’s modulus exceeding those of the nanofiber mats.

## 3. Results and Discussion

**Figure 1:**
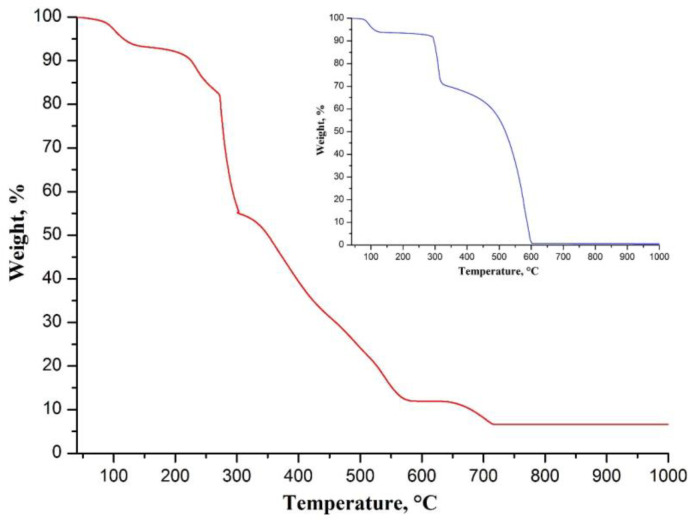
TG curve of electrospun CaAA/PAN fibers. The insert shows TG curve of electrospun PAN fibers.

**Figure 2:**
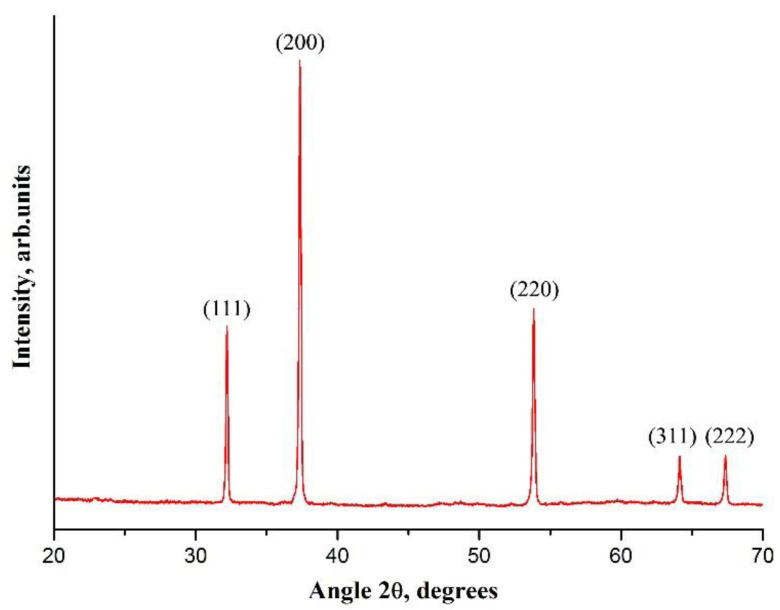
The XRD patterns of electrospun CaAA/PAN fibers annealed at 800 °C.

Figure 1 shows the results of the TG analysis of the electrospun composite fibers. The first step of weight loss of 6.7 wt.% before 148 °C is due to the evaporation of the residual solvent from the filaments. The main weight drop of 81.4 wt.% is observed between 148 and 581 °C and corresponds to the stabilization of PAN and its combustion at the elevated temperatures in an oxygen-containing atmosphere (insert of Figure 1) and the decomposition of CaAA resulting in the formation of CaCO_3_ [22]. The stabilization of PAN is a multistage process that occurs at about 300 °C and includes the cyclization of the nitrile groups and cross-linking of the chain molecules, followed by dehydrogenation and oxidative reactions [23]. The polymer completely burns out near 600 °C and the filaments contain only CaCO_3_ up to 630 °C, since the last weight decrease from 11.9 to 6.6 wt.% between 630 and 717 °C corresponds to the transformation of CaCO_3_ to CaO. Thus, the electrospun CaAA/PAN fibers become CaO fibers if they are annealed at temperatures exceeding 717 °C, which is confirmed by the plateau in the TG curve (Figure 1) and the obtained XRD results (Figure 2).

Figure 2 shows the XRD pattern of CaAA/PAN fibers annealed at 800 °C. It indicates that the fabricated filaments have crystalline structure and are composed only of CaO grains, since the observed reflections at 32.2°, 37.3°, 53.8°, 64.1° and 67.3° are the characteristic peaks of CaO. The average CaO grain size is estimated as 74 nm. The absence of the peaks near 24° and 43°, which are associated with the crystallographic planes of graphene [24], indicates that no carbon-containing fibers are formed when CaAA/PAN filaments are annealed at elevated temperatures in air. To be carbonized, the pre-stabilized PAN-containing fibers must be heat-treated at high temperatures in an inert atmosphere to prevent polymer burnout [25].

**Figure 3:**
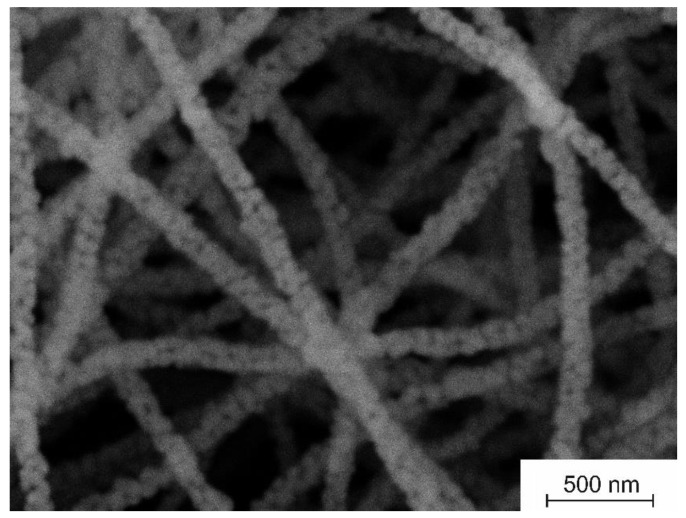
The microstructure of electrospun CaAA/PAN fibers annealed at 800 °C.

The grain structure of the prepared CaO fibers is confirmed by SEM microstructural analysis (Figure 3). The resulting filaments are nanofibers, which are characterized by the coarse surface and the average diameter of 130 ± 11 nm. Since nanofibers contain no binding polymer and no products of its thermal decomposition, and the sintering of CaO grains does not occur at 800 °C because the Tammann temperature of CaO is 1313 °C [26], we suppose that only the Van der Waals forces provide the connection between CaO grains inside the nanofibers.

It can be clearly seen in Figure 3 that the fabricated nanofibers are porous. The pores of arbitrary size are located at the junctions of adjacent CaO grains. The nitrogen adsorption measurements indicate that the specific surface area and the specific pore volume of CaO nanofibers are 31 m^2^/g and 0.051 cm^3^/g, respectively. Previously, zirconia nanofibers with the average diameter of about 100 nm and close values of specific surface area and specific pore volume were fabricated from the electrospun composite filaments containing zirconia acetylacetonate and polyacrylonitrile [27].

**Figure 4:**
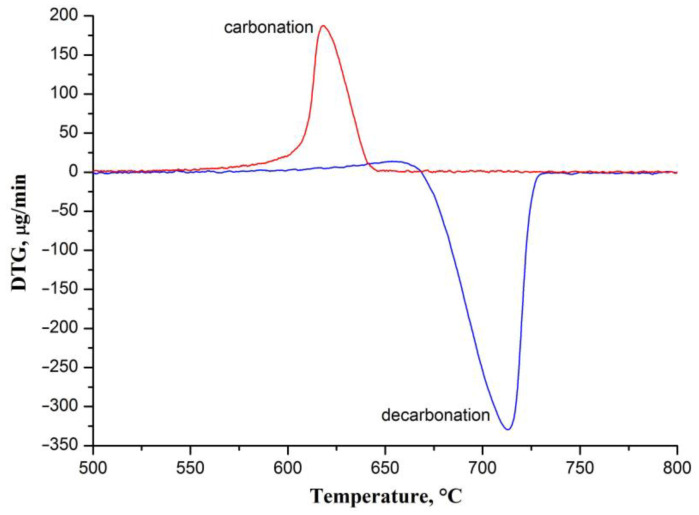
The DTG curves of CaO nanofibers carbonation and decarbonation processes.

According to the performed DTG analysis, CaO nanofibers most intensively sorb CO_2_ in the temperature range of 610–633 °C, which is determined by the carbonation-rate peak width at half height (Figure 4). The carbonation rate attains the maximum value at 618 °C. Therefore, the given temperature was chosen as the carbonation temperature.

**Figure 5:**
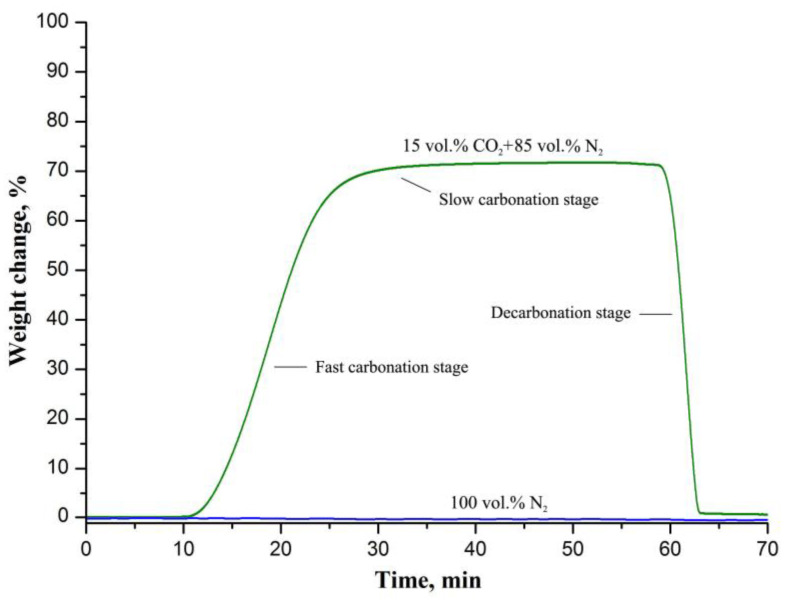
The carbonation–decarbonation cycle profiles of CaO nanofibers upon carbonation in the gaseous stream containing 15 vol.% CO_2_ and 85 vol.% N_2_; and in pure N_2_.

Figure 5 shows the carbonation–decarbonation cycle profile in the temperature range of 500–800 °C.

During carbonation the mass of CaO nanofibers increases by 72.15 wt.% that corresponds to the CO_2_-uptake capacity of 16.4 mmol/g. The fabricated CaO nanofibers show a rather high capacity since the stoichiometric capacity of CaO is 17.9 mmol/g. The observed absence of a change in the weight of the sorbent upon carbonation in pure N_2_ indicates that it does not sorb N_2_ in the temperature range of 500–800 °C. The CO_2_-uptake capacity of CaO nanofibers is inferior to that of the nanosized powder CaO sorbent that was fabricated using flame-spray pyrolysis [10] and exceeds, for example, the capacity of nanosized powder CaO sorbents produced by the calcination method [28], the sol-gel technique [29] and mechanical milling [12]. The characteristics of synthetic CaO sorbents produced by various methods are presented in Table 1.

Table 1 shows that both the synthesis method and the microstructure of the sorbent affect its performance. However, it can be concluded that CO_2_-uptake capacity of the sorbent increases with the rise in its specific surface area and porosity. We suppose that the regular macroporous structure and small fiber diameter improve CO_2_-uptake capacity of the electrospun CaO sorbent.

The carbonation process includes two stages (Figure 5). A fast initial chemical-controlled stage is followed by a slower diffusion-controlled stage once a layer of carbonate has formed on the surface of the calcium oxide particles [30]. A product layer impedes CO_2_ transport inside the CaO particles. In our case, the carbonation reaction is essentially chemical-controlled, since the slow carbonation stage is barely discernible in Figure 5. This is probably due to the small size of the CaO grains that form the nanofibers. In [31] it was calculated that the carbonation reaction follows a slower diffusion-controlled stage when the product layer reaches a thickness of about 50 nm. Therefore, when the CaO particle size less is than 50 nm, only the chemical-controlled stage occurs. The regular macroporous structure of the electrospun fiber mat ensures efficient gas transport (Figure 3) and, as a result, improves the capacity of the sorbent.

In [32] it was revealed that the increased decarbonation temperature negatively affects the capacity of CaO, especially under a multi-cycle carbonation–decarbonation process, due to CaCO_3_ sintering, which occurs not only during carbonation but also during heating to the decarbonation temperature and upon decarbonation. The reason is the low Tammann temperature of CaCO_3_ (533 °C) [26]. In accordance with the obtained DTG data (Figure 4), the CaO nanofibers most intensively desorb CO_2_ in the temperature range of 690–721 °C (half-height width of the decarbonation-rate peak), and the decarbonation rate is at a maximum at 713 °C. Thus, nanofibrous CaO can be decarbonized at a lower temperature than the 800 °C that was used. Typically, CaO-based sorbents are characterized by a decarbonation temperature in the range of 850–950 °C [30]. A lower regeneration temperature is preferred for temperature swing adsorption processes and, in particular, the calcium-looping process due to reduced energy consumption.

**Figure 6:**
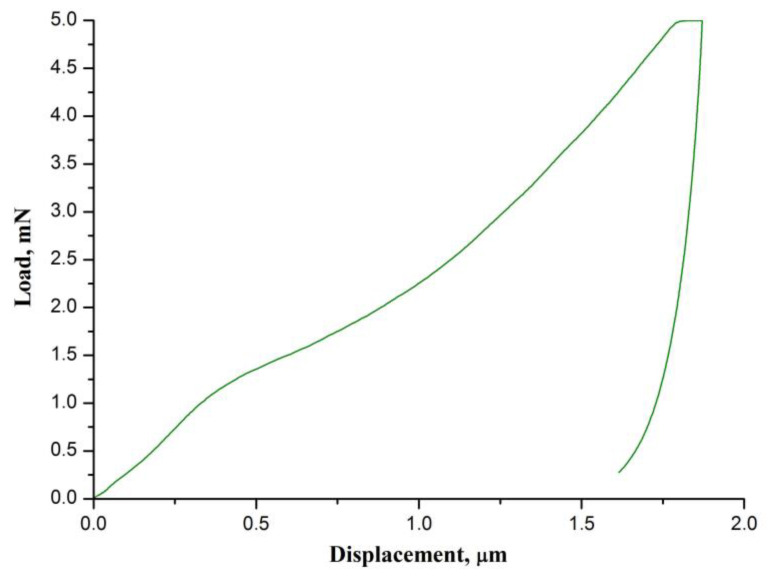
The normalized DTG signals of CaO nanofiber carbonation and decarbonation processes.

Figure 6 illustrates the load–displacement curve obtained for the fabricated CaO nanofiber mat.

The calculated values of hardness and the indentation Young’s modulus are 1.87 ± 0.05 MPa and 786 ± 91 MPa, respectively. The hardness and Young’s modulus of the CaO nanofiber mats are higher than those of yttria-stabilized zirconia nanofiber mats obtained at 700–900 °C (hardness of about 1 MPa and Young’s modulus of about 150 MPa) [27]. It indicates that the CaO nanofiber mats are more brittle and stiffer than the 3 mol% Y_2_O_3_-ZrO_2_ nanofiber mats, which were prepared at the same temperature. We assume that the reason is the larger size of the CaO grains, since the larger size hinders the grains’ free movement in the nanofibers. The nanofibers become stiffer and their Young’s modulus increases. As a result, the Young’s modulus of the mat also increases. The obtained CaO nanofiber mats are also stiffer than the TiO_2_ nanofiber mats (Young’s modulus of 461 MPa) reported in [33].

## 4. Conclusions

For the first time, the CaO-based sorbent for high-temperature CO_2_ capture was fabricated using the electrospinning technique. The calcination at 800 °C of electrospun composite filaments containing calcium acetylacetonate and polyacrylonitrile as a calcium-oxide precursor and a binder polymer, respectively, resulted in a CaO nanofibrous mat with a specific surface area of 31 m^2^/g, a CO_2_-uptake capacity of 16.4 mmol/g at the carbonation temperature of 618 °C, a hardness of 1.87 MPa and the indentation Young’s modulus of 786 MPa. The decarbonation temperature below 740 °C makes the CaO nanofiber sorbent promising for CO_2_ removal from hot gas streams by the temperature-swing adsorption technology.

## Figures and Tables

**Table 1:** Comparison of synthetic CaO sorbents obtained by various methods.

Method	CO_2_-Uptake Capacity, mmol/g	Specific Surface Area, m^2^/g	Specific Pore Volume, cm^3^/g	Reference
Electrospinning	16.4	31	0.051	present work
Flame-spray pyrolysis	17.1	46	0.170	[10]
Calcination method	15.7	17	0.271	[28]
Sol-gel method	13.2	45	0.080	[29]
Mechanical milling	6.6	19	0.064	[12]

## Data Availability

All data included in this study are available upon request from the corresponding author.
